# 6-(4-Nitro­benz­yloxy)quinoline

**DOI:** 10.1107/S1600536809025823

**Published:** 2009-07-08

**Authors:** Min Min Zhao, Yong Hua Li, Yuan Zhang

**Affiliations:** a Ordered Matter Science Research Center, College of Chemistry and Chemical Engineering, Southeast University, Nanjing 211189, People’s Republic of China

## Abstract

In the mol­ecule of the title compound, C_16_H_12_N_2_O_3_, the nitrobenzene benzene ring forms a dihedral angle of 23.8 (8)° with the plane of the quinoline ring system. The crystal structure is stabilized by an aromatic π–π stacking inter­action between centrosymmetrically related benzene rings [centroid–centroid distance 3.663 (2) Å].

## Related literature

For related structures, see: Fu & Zhao (2007 ▶); Li & Chen (2008 ▶); Zhao (2008 ▶); Zhao *et al.* (2009 ▶).
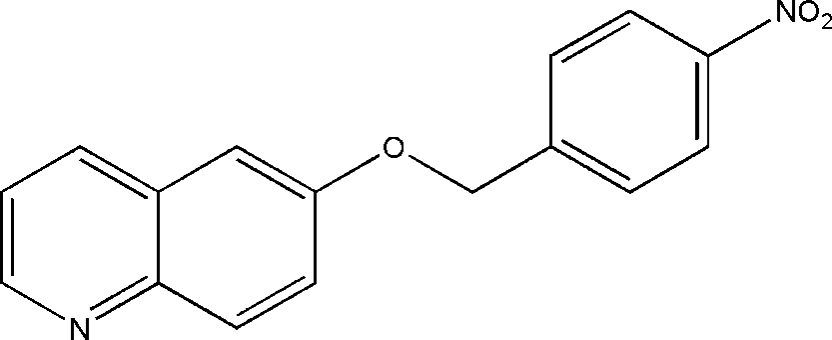


## Experimental

### 

#### Crystal data

C_16_H_12_N_2_O_3_
*M* *_r_* = 280.28
Monoclinic, 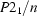

*a* = 12.296 (3) Å
*b* = 8.9146 (18) Å
*c* = 13.559 (3) Å
β = 115.25 (3)°
*V* = 1344.3 (6) Å^3^
*Z* = 4
Mo *K*α radiation
μ = 0.10 mm^−1^
*T* = 293 K
0.20 × 0.18 × 0.15 mm

#### Data collection

Rigaku SCXmini diffractometer
Absorption correction: multi-scan (*CrystalClear*; Rigaku, 2005 ▶) *T*_min_ = 0.976, *T*_max_ = 0.981
11965 measured reflections
2630 independent reflections
1372 reflections with *I* > 2σ(*I*)
*R*_int_ = 0.079

#### Refinement

*R*[*F* ^2^ > 2σ(*F* ^2^)] = 0.065
*wR*(*F* ^2^) = 0.152
*S* = 1.02
2630 reflections
190 parameters
H-atom parameters constrained
Δρ_max_ = 0.15 e Å^−3^
Δρ_min_ = −0.15 e Å^−3^

### 

Data collection: *CrystalClear* (Rigaku, 2005 ▶); cell refinement: *CrystalClear*; data reduction: *CrystalClear*; program(s) used to solve structure: *SHELXS97* (Sheldrick, 2008 ▶); program(s) used to refine structure: *SHELXL97* (Sheldrick, 2008 ▶); molecular graphics: *SHELXTL/PC* (Sheldrick, 2008 ▶); software used to prepare material for publication: *SHELXTL/PC*.

## Supplementary Material

Crystal structure: contains datablocks I, global. DOI: 10.1107/S1600536809025823/rz2340sup1.cif

Structure factors: contains datablocks I. DOI: 10.1107/S1600536809025823/rz2340Isup2.hkl

Additional supplementary materials:  crystallographic information; 3D view; checkCIF report

## Figures and Tables

**Fig. 1.**
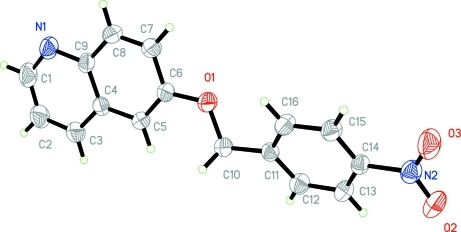
The molecular structure of the title compound with the atom numbering scheme. Displacement ellipsoids are drawn at the 30% probability level.

## supplementary crystallographic information

### Comment

Recently, we have reported the synthesis and crystal structure of some benzonitrile compounds (Fu & Zhao, 2007; Li & Chen, 2008; Zhao, 2008; Zhao *et al.*, 2009). As an extension of our work on the structural characterization of benzonitrile derivatives, we present herein the synthesis and crystal structure of the title compound.

In the molecule of the title compound (Fig. 1), bond lengths and angles are within normal ranges. The C11–C16 benzene ring forms a dihedral angle of 23.8 (8)° with the plane of the quinoline ring system. No intra- or intermolecular hydrogen bonds are observed. The crystal structure is stabilized by an aromatic π-π stacking interaction involving centrosymmetrically related benzene rings at (x, y, z) and (1-x, -y, 1-z), with a centroid-centroid distance of 3.663 (2) Å.

### Experimental

Quinolin-6-ol (1 g,0.0069 mol) was added to a solution of sodium hydroxide (0.276 g, 0.0069 mol) in methanol (15 ml) and stirred for one hour. Then 4-(bromomethyl)benzonitrile (1.49 g, 0.0069 mol) was added and the mixture stirred at room temperature for 1 day. The title compound was isolated by column chromatography using petroleum ether/ethyl acetate (1:1 v/v) as eluent. Single crystals suitable for X-ray diffraction analysis were obtained by slow evaporation of an ethyl acetate/tetrahydrofuran (3:1 v/v) solution.

### Refinement

All H atoms were calculated geometrically allowed to ride on their parent atoms, with C—H = 0.93-0.97 Å, and with *U*_iso_(H) = 1.2U_eq_ (C).

### Crystal data

**Table:**

C_16_H_12_N_2_O_3_	*F*(000) = 584
*M**_r_* = 280.28	*D*_x_ = 1.385 Mg m^−^^3^
Monoclinic, *P*2_1_/*n*	Mo *K*α radiation, λ = 0.71073 Å
Hall symbol: -P 2yn	Cell parameters from 8689 reflections
*a* = 12.296 (3) Å	θ = 3.0–27.8°
*b* = 8.9146 (18) Å	µ = 0.10 mm^−^^1^
*c* = 13.559 (3) Å	*T* = 293 K
β = 115.25 (3)°	Block, colourless
*V* = 1344.3 (6) Å^3^	0.20 × 0.18 × 0.15 mm
*Z* = 4	

### Data collection

**Table:**

Rigaku SCXmini diffractometer	2630 independent reflections
Radiation source: fine-focus sealed tube	1372 reflections with *I* > 2σ(*I*)
graphite	*R*_int_ = 0.079
Detector resolution: 13.6612 pixels mm^-1^	θ_max_ = 26.0°, θ_min_ = 3.3°
CCD profile fitting scans	*h* = −15→15
Absorption correction: multi-scan (*CrystalClear*; Rigaku, 2005)	*k* = −10→10
*T*_min_ = 0.976, *T*_max_ = 0.981	*l* = −16→16
11965 measured reflections	

### Refinement

**Table:**

Refinement on *F*^2^	Primary atom site location: structure-invariant direct methods
Least-squares matrix: full	Secondary atom site location: difference Fourier map
*R*[*F*^2^ > 2σ(*F*^2^)] = 0.065	Hydrogen site location: inferred from neighbouring sites
*wR*(*F*^2^) = 0.152	H-atom parameters constrained
*S* = 1.02	*w* = 1/[σ^2^(*F*_o_^2^) + (0.0563*P*)^2^ + 0.1856*P*] where *P* = (*F*_o_^2^ + 2*F*_c_^2^)/3
2630 reflections	(Δ/σ)_max_ < 0.001
190 parameters	Δρ_max_ = 0.14 e Å^−^^3^
0 restraints	Δρ_min_ = −0.15 e Å^−^^3^

### Special details

**Table:**

Geometry. All e.s.d.'s (except the e.s.d. in the dihedral angle between two l.s. planes) are estimated using the full covariance matrix. The cell e.s.d.'s are taken into account individually in the estimation of e.s.d.'s in distances, angles and torsion angles; correlations between e.s.d.'s in cell parameters are only used when they are defined by crystal symmetry. An approximate (isotropic) treatment of cell e.s.d.'s is used for estimating e.s.d.'s involving l.s. planes.
Refinement. Refinement of *F*^2^ against ALL reflections. The weighted *R*-factor *wR* and goodness of fit *S* are based on *F*^2^, conventional *R*-factors *R* are based on *F*, with *F* set to zero for negative *F*^2^. The threshold expression of *F*^2^ > σ(*F*^2^) is used only for calculating *R*-factors(gt) *etc*. and is not relevant to the choice of reflections for refinement. *R*-factors based on *F*^2^ are statistically about twice as large as those based on *F*, and *R*- factors based on ALL data will be even larger.

### Fractional atomic coordinates and isotropic or equivalent isotropic displacement parameters (Å^2^)

**Table:**

	*x*	*y*	*z*	*U*_iso_*/*U*_eq_	
C1	0.3786 (4)	0.3414 (4)	−0.1994 (3)	0.0901 (11)	
H1A	0.3497	0.3839	−0.2686	0.108*	
C2	0.4967 (4)	0.3728 (4)	−0.1275 (3)	0.0881 (11)	
H2A	0.5444	0.4336	−0.1487	0.106*	
C3	0.5411 (3)	0.3132 (4)	−0.0257 (3)	0.0735 (9)	
H3A	0.6202	0.3321	0.0235	0.088*	
C4	0.4671 (3)	0.2230 (3)	0.0046 (2)	0.0542 (7)	
C5	0.5069 (2)	0.1580 (3)	0.1090 (2)	0.0557 (7)	
H5A	0.5847	0.1750	0.1616	0.067*	
C6	0.4303 (3)	0.0705 (3)	0.1319 (2)	0.0555 (8)	
C7	0.3126 (3)	0.0454 (3)	0.0539 (2)	0.0657 (8)	
H7A	0.2613	−0.0147	0.0711	0.079*	
C8	0.2730 (3)	0.1074 (3)	−0.0460 (2)	0.0650 (8)	
H8A	0.1943	0.0902	−0.0967	0.078*	
C9	0.3483 (3)	0.1972 (3)	−0.0747 (2)	0.0572 (8)	
C10	0.5763 (2)	0.0164 (3)	0.3124 (2)	0.0597 (8)	
H10A	0.5943	0.1212	0.3318	0.072*	
H10B	0.6332	−0.0211	0.2861	0.072*	
C11	0.5866 (2)	−0.0718 (3)	0.4102 (2)	0.0502 (7)	
C12	0.6722 (3)	−0.0323 (3)	0.5119 (2)	0.0620 (8)	
H12A	0.7211	0.0504	0.5189	0.074*	
C13	0.6870 (3)	−0.1124 (3)	0.6032 (2)	0.0605 (8)	
H13A	0.7456	−0.0855	0.6715	0.073*	
C14	0.6139 (2)	−0.2321 (3)	0.5916 (2)	0.0504 (7)	
C15	0.5270 (3)	−0.2749 (3)	0.4919 (2)	0.0607 (8)	
H15A	0.4777	−0.3568	0.4859	0.073*	
C16	0.5142 (3)	−0.1945 (3)	0.4008 (2)	0.0627 (8)	
H16A	0.4565	−0.2231	0.3325	0.075*	
N1	0.3048 (2)	0.2567 (3)	−0.1773 (2)	0.0782 (8)	
N2	0.6285 (3)	−0.3179 (3)	0.6883 (2)	0.0655 (7)	
O1	0.45682 (16)	0.0016 (2)	0.22964 (15)	0.0699 (6)	
O2	0.7111 (2)	−0.2867 (3)	0.77502 (18)	0.0885 (8)	
O3	0.5578 (2)	−0.4184 (3)	0.67829 (19)	0.1026 (9)	

### Atomic displacement parameters (Å^2^)

**Table:**

	*U*^11^	*U*^22^	*U*^33^	*U*^12^	*U*^13^	*U*^23^
C1	0.105 (3)	0.111 (3)	0.062 (2)	0.029 (3)	0.043 (2)	0.032 (2)
C2	0.096 (3)	0.101 (3)	0.080 (3)	0.005 (2)	0.050 (2)	0.028 (2)
C3	0.074 (2)	0.083 (2)	0.070 (2)	0.0011 (18)	0.0365 (18)	0.0121 (19)
C4	0.0594 (18)	0.0558 (17)	0.0529 (17)	0.0062 (14)	0.0293 (15)	0.0049 (15)
C5	0.0499 (17)	0.0636 (19)	0.0504 (17)	0.0014 (14)	0.0182 (14)	0.0028 (15)
C6	0.0569 (18)	0.0606 (19)	0.0488 (17)	0.0038 (15)	0.0224 (15)	0.0096 (15)
C7	0.0593 (19)	0.075 (2)	0.061 (2)	−0.0085 (16)	0.0246 (17)	0.0075 (17)
C8	0.0535 (18)	0.078 (2)	0.0564 (19)	−0.0016 (16)	0.0167 (16)	−0.0063 (17)
C9	0.0624 (19)	0.064 (2)	0.0458 (17)	0.0152 (16)	0.0241 (15)	0.0030 (15)
C10	0.0556 (18)	0.0685 (19)	0.0551 (17)	−0.0006 (15)	0.0235 (15)	0.0073 (16)
C11	0.0513 (16)	0.0530 (17)	0.0498 (17)	0.0060 (14)	0.0249 (14)	0.0037 (14)
C12	0.065 (2)	0.0585 (19)	0.0581 (19)	−0.0059 (15)	0.0220 (16)	−0.0016 (16)
C13	0.0649 (19)	0.063 (2)	0.0443 (17)	0.0002 (16)	0.0144 (15)	−0.0018 (15)
C14	0.0552 (17)	0.0486 (17)	0.0475 (17)	0.0149 (14)	0.0219 (14)	0.0077 (14)
C15	0.0610 (18)	0.0558 (18)	0.0592 (19)	−0.0042 (14)	0.0196 (16)	0.0100 (16)
C16	0.0626 (19)	0.064 (2)	0.0515 (17)	−0.0022 (16)	0.0150 (15)	0.0042 (16)
N1	0.081 (2)	0.097 (2)	0.0538 (16)	0.0222 (16)	0.0264 (15)	0.0179 (15)
N2	0.0721 (18)	0.0630 (18)	0.0608 (18)	0.0157 (15)	0.0278 (15)	0.0106 (15)
O1	0.0584 (13)	0.0896 (15)	0.0573 (13)	−0.0055 (11)	0.0204 (11)	0.0238 (11)
O2	0.0970 (18)	0.0955 (18)	0.0544 (13)	0.0180 (14)	0.0145 (13)	0.0151 (13)
O3	0.114 (2)	0.106 (2)	0.0789 (17)	−0.0218 (17)	0.0334 (15)	0.0297 (15)

### Geometric parameters (Å, °)

**Table:**

C1—N1	1.310 (4)	C10—O1	1.425 (3)
C1—C2	1.389 (4)	C10—C11	1.500 (3)
C1—H1A	0.9300	C10—H10A	0.9700
C2—C3	1.358 (4)	C10—H10B	0.9700
C2—H2A	0.9300	C11—C12	1.377 (4)
C3—C4	1.402 (4)	C11—C16	1.382 (4)
C3—H3A	0.9300	C12—C13	1.373 (4)
C4—C5	1.410 (4)	C12—H12A	0.9300
C4—C9	1.415 (4)	C13—C14	1.360 (4)
C5—C6	1.357 (4)	C13—H13A	0.9300
C5—H5A	0.9300	C14—C15	1.372 (4)
C6—O1	1.368 (3)	C14—N2	1.461 (3)
C6—C7	1.399 (4)	C15—C16	1.378 (4)
C7—C8	1.346 (4)	C15—H15A	0.9300
C7—H7A	0.9300	C16—H16A	0.9300
C8—C9	1.399 (4)	N2—O2	1.214 (3)
C8—H8A	0.9300	N2—O3	1.215 (3)
C9—N1	1.367 (3)		
			
N1—C1—C2	125.1 (3)	O1—C10—H10A	110.0
N1—C1—H1A	117.5	C11—C10—H10A	110.0
C2—C1—H1A	117.5	O1—C10—H10B	110.0
C3—C2—C1	118.8 (3)	C11—C10—H10B	110.0
C3—C2—H2A	120.6	H10A—C10—H10B	108.4
C1—C2—H2A	120.6	C12—C11—C16	118.8 (3)
C2—C3—C4	119.5 (3)	C12—C11—C10	119.4 (3)
C2—C3—H3A	120.2	C16—C11—C10	121.7 (3)
C4—C3—H3A	120.2	C13—C12—C11	121.4 (3)
C3—C4—C5	122.6 (3)	C13—C12—H12A	119.3
C3—C4—C9	117.4 (3)	C11—C12—H12A	119.3
C5—C4—C9	120.0 (3)	C14—C13—C12	118.5 (3)
C6—C5—C4	119.2 (3)	C14—C13—H13A	120.8
C6—C5—H5A	120.4	C12—C13—H13A	120.8
C4—C5—H5A	120.4	C13—C14—C15	122.1 (3)
C5—C6—O1	125.3 (3)	C13—C14—N2	119.0 (3)
C5—C6—C7	120.9 (3)	C15—C14—N2	119.0 (3)
O1—C6—C7	113.8 (3)	C14—C15—C16	118.8 (3)
C8—C7—C6	120.6 (3)	C14—C15—H15A	120.6
C8—C7—H7A	119.7	C16—C15—H15A	120.6
C6—C7—H7A	119.7	C15—C16—C11	120.4 (3)
C7—C8—C9	121.1 (3)	C15—C16—H16A	119.8
C7—C8—H8A	119.5	C11—C16—H16A	119.8
C9—C8—H8A	119.5	C1—N1—C9	116.6 (3)
N1—C9—C8	119.2 (3)	O2—N2—O3	122.7 (3)
N1—C9—C4	122.6 (3)	O2—N2—C14	118.8 (3)
C8—C9—C4	118.2 (3)	O3—N2—C14	118.5 (3)
O1—C10—C11	108.5 (2)	C6—O1—C10	117.5 (2)
